# Antiangiogenic molecules from marine actinomycetes and the importance of using zebrafish model in cancer research

**DOI:** 10.1016/j.heliyon.2020.e05662

**Published:** 2020-12-07

**Authors:** Jhansi Nathan, Rajaretinam Rajesh Kannan

**Affiliations:** a AUKBC Research Centre, Anna University, MIT Campus, Chromepet, Chennai 600044, Tamil Nadu, India; b Centre for Molecular and Nanomedical Sciences, Centre for Nanoscience and Nanotechnology, School of Bio and Chemical Engineering, Sathyabama Institute of Science and Technology (Deemed to be University), Chennai 600119, India

**Keywords:** *Danio rerio*, Angiogenesis, Actinomycetes, Bioactive molecules, Transgenic model, Xenograft model, Biotechnology, Genetics, Proteins, Pharmaceutical science, Biomedical engineering, Molecular biology, Cancer research, Developmental biology, Toxicology

## Abstract

Blood vessel sprouting from pre-existing vessels or angiogenesis plays a significant role in tumour progression. Development of novel biomolecules from marine natural sources has a promising role in drug discovery specifically in the area of antiangiogenic chemotherapeutics. Symbiotic actinomycetes from marine origin proved to be potent and valuable sources of antiangiogenic compounds. Zebrafish represent a well-established model for small molecular screening and employed to study tumour angiogenesis over the last decade. Use of zebrafish has increased in the laboratory due to its various advantages like rapid embryo development, optically transparent embryos, large clutch size of embryos and most importantly high genetic conservation comparable to humans. Zebrafish also shares similar physiopathology of tumour angiogenesis with humans and with these advantages, zebrafish has become a popular model in the past decade to study on angiogenesis related disorders like diabetic retinopathy and cancer. This review focuses on the importance of antiangiogenic compounds from marine actinomycetes and utility of zebrafish in cancer angiogenesis research.

## 1 Introduction

**Figure 1:**
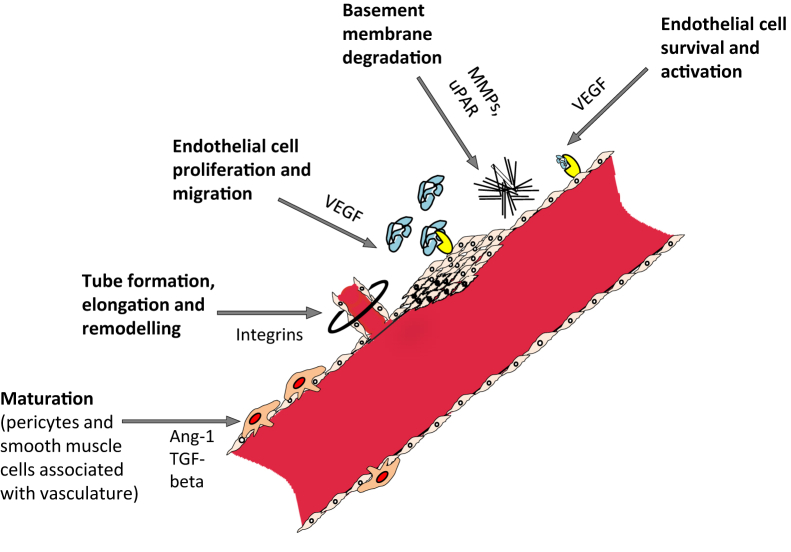
The process of angiogenesis.

**Figure 2:**
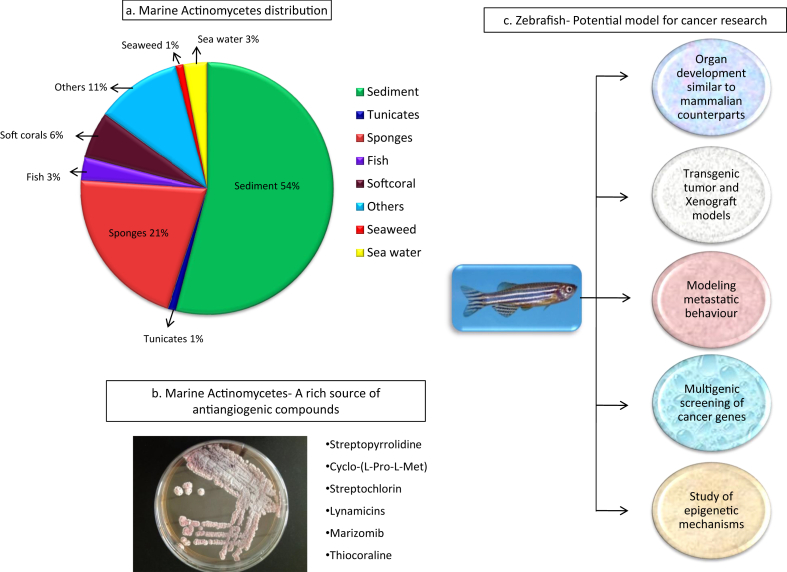
Importance of zebrafish model to study antiangiogenic compounds from marine actinomycetes (a. Distribution of actinomycetes from marine sources [14], b. Antiangiogenic biomolecules from marine actinomycetes, c. Advantages of zebrafish model in cancer research).

Blood vessels' sprouting from preexisting vasculature is angiogenesis, which can occur at both physiological as well as pathological conditions like wound healing, placentation, embryogenesis, inflammatory disorders and tumour growth [1, 2]. In tumour angiogenesis, tumour cell releases certain molecules that signal the host tissue and activate specific genes to make protein that boost development of novel blood vessels (Figure 1) [3]. Vascular endothelial growth factor (VEGF) is the key angiogenic determinant factor of angiogenesis (VEGF) and targeting its expression; thereby blocking the VEGF signaling cascade would be significantly useful in the development of new anticancer drugs [4]. Many novel bioactive molecules from natural sources are undergoing clinical trials to downregulate vegf and thereby disrupt the growth of angiogenic vessels [5, 6]. Natural products from marine sources are increasing popularity in drug discovery, especially the marine actinobacteria plays major role in development of novel bioactive compounds. The idea of employing marine bioactive molecules to target angiogenic growth factors has been of a great importance in the past three decades after the substantial contribution by Dr Judah Folkman [7]. Marine invertebrates such as molluscs, gorgonia, soft coral, sponges, sponge-associated bacteria and actinomycetes, have been widely explored for possible angiogenic inhibitors [8]. Small molecular compounds from marine origin have become important in cancer research as well as in the study of antibacterial, antifungal, antiviral and anti-coagulant properties [9, 10]. It is evident that conventional treatment for cancer has many side effects and it is crucial to develop natural products based anticancer therapies in future. In angiogenic drug discovery efforts, rodent models have dominated to date, however, these models are not suitable for large-scale drug screening when compared to the advantages of zebrafish which requires minimal labor, resources and time. Furthermore, ethical issues in the usage of rodents have made their usage even more limited [11, 12, 13]. Zebrafish is an extensive model organism to study small molecular drug interactions as it provides a series of advantages like optical transparency, rapid development, and high number of offspring. Marine actinomycetes are distributed widely and thus, discovering novel antiangiogenic compounds from them can serve as promising candidates for cancer drug discovery. Figure 2 depicts the distribution of actinomycetes from marine sources, antiangiogenic small molecules discovered from marine actinomycetes and the importance of utilizing zebrafish in cancer research [14]. Thus, this review focuses on the utilization of zebrafish as a relevant model organism in antiangiogenic drug discovery mainly about marine symbiotic actinomycetes and drug screens.

## 2 Antiangiogenic agents from marine actinomycetes

**Figure 3:**
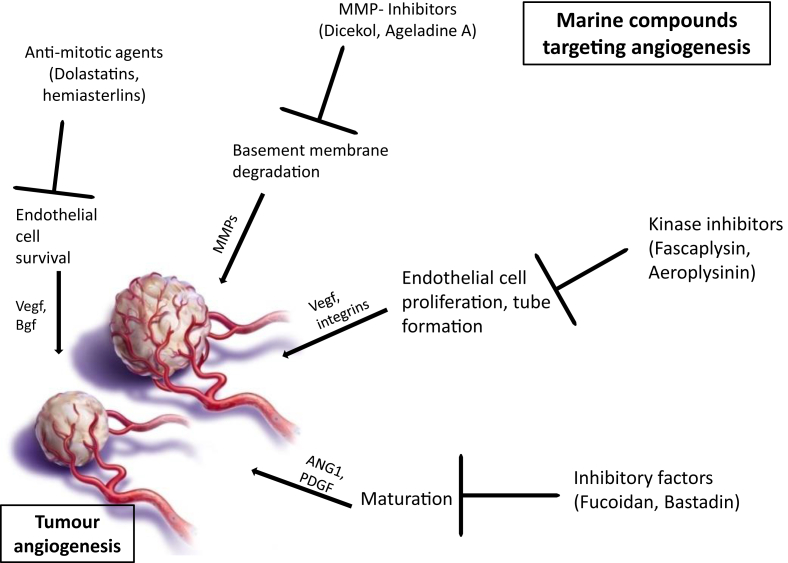
Marine derived drugs targeting tumour angiogenesis.

Marine sources are rich in secondary metabolites and there are many compounds reported to possess anticancer properties. In a recent review, it has been elucidated that more than 45 compounds from marine origin are shown to have antiangiogenic potential and 10 of them have already entered clinical trials at different phases for cancer therapy [8]. These compounds include terpenes, saccharides, saponins, macrocycles, xanthones, peptides, alkaloids and pyrones which display a great structural and chemical diversity and also these compounds downregulate angiogenesis by altering distinct targets due to their unique structures. These angiogenesis inhibitors act directly on the endothelial cells or other growth factors of the angiogenic cascade (Figure 3) and they hinder the growth of the endothelial cells by arresting the cell cycle during mitosis or by causing DNA damage leading to apoptosis [15].

**Figure 4:**
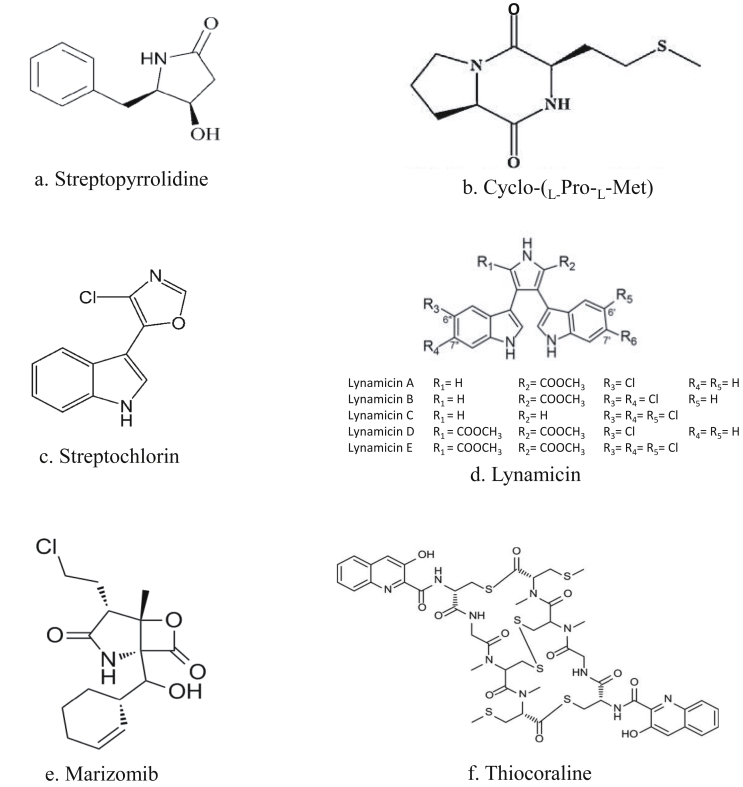
Structures of marine actinomycetes derived compounds that possess antiangiogenic potential.

Actinomycetes are filamentous Gram-positive bacteria which belongs to the phylum Actinobateria, are considered to be the largest group in the bacterial domain [16]. Bioactive molecules from actinomycetes are reported to be the highest among the other bacterial species which is almost 45 percent of the overall metabolites reported [17]. Streptomyces is the major group among actinomycetes which has produced around 7,600 compounds [17] and they have also produced clinically important antitumour agents [18, 19]. The undesirable side effects and high toxicity of already available chemotherapy drugs for cancer treatment makes the researches to discover novel antitumour drugs from marine origin or phytochemicals which have no/less side effects when compared to conventional therapy [20]. Marine actinomycetes are unique in producing secondary metabolites when compared to other microorganisms from terrestrial origin; with many pharmacologically important activities like anti-oxidant, anti-inflammation and antitumour properties [21, 22, 23, 24, 25]. Table 1 lists some of the important antitumour compounds from marine actinomycetes. Marine microorganisms possess unique features and thus, they might synthesize different secondary metabolites in their challenging habitats [100]. Most important derivatives from marine actinomycetes which possess antiangiogenic potential are described in Table 2 and their structures are shown in Figure 4.

**Table 1:** Antitumour compounds produced by marine actinomycetes.

Structural type	Compound	Organism	Reference
Indole	3,6-disubstituted indoles	*Streptomyces* sp. BL-49-58-005	[26]
Indole	Streptochlorin	*Streptomyces* sp. 04DH110	[27, 28, 29, 30]
Polyketide	1-hydroxy-1-norresistomycin	*Streptomyces chinaensis* AUBN1/7 *Streptomyces* sp. B8005	[31, 32]
Polyketide	1,8-dihydroxy-2-ethyl-3-Methylanthraquinone	*Streptomyces* sp. FX-58	[33]
Polyketide	Actinofuranones	*Streptomyces* sp. CNQ766	[34]
Polyketide	Arenicolides	*Salinispora arenicola* CNR-005	[35]
Polyketide	Aureoverticillactam	*Streptomyces aureoverticillatus* NPS001583	[36]
Polyketide	Chalcomycin	*Streptomyces* sp. M491	[37]
Polyketide	Chalcomycin B	*Streptomyces* sp. B7064 *Streptomyces* sp M491	[37, 38]
Polyketide	Chartreusin	*Streptomyces* sp.QD518	[39]
Polyketide	Cyanosporasides	*Salinispora pacifica* CNS103	[40]
Polyketide	Daryamides	*Streptomyces* sp. CNQ-085	[41]
Polyketide	Fridamycin D	*Streptomyces* sp. B6921	[42]
Polyketide	Griseorhodin A	*Streptomyces* sp. JP95	[43, 44]
Polyketide	Himalomycins	*Streptomyces* sp. B6921	[42]
Polyketide	IB-0028	*Actinomadura* sp. BL-42-PO13-046	[45, 46]
Polyketide	IB-96212	*Micromonospora* sp. L-25-ES25-008	[47, 48]
Polyketide	Komodoquinones	*Streptomyces* sp. KS3	[49, 50]
Polyketide	Manumycin A	*Streptomyces* sp. M045	[51]
Polyketide	Marinomycins	*Marinispora* sp. CNQ-140	[52]
Polyketide	Marmycins	*Streptomyces* sp. CNH990	[53]
Polyketide	Nonactin	*Streptomyces* sp. KORDI-3238	[54]
Polyketide	Pacificanones	*Salinispora pacifica* CNS-237	[55]
Polyketide	Parimycin	*Streptomyces* sp. B8652	[56]
Polyketide	Piericidins	*Streptomyces* sp. YM14-060	[57, 58]
Polyketide	Rabelomycin	*Streptomyces* sp. B6921	[42]
Polyketide	Resitoflavine	*Streptomyces chinaensis* AUBN1/7 *Streptomyces* sp. B8005	[31, 32, 59]
Polyketide	Resistomycin	*Streptomyces* sp. B8005 *Streptomyces* sp. B4842	[32]
Polyketide	Saliniketals	*Salinispora arenicola* CNR-005	[60]
Polyketide	Salinipyrones	*Salinispora pacifica* CNS-237	[55]
Polyketide	Sporolides	*Salinispora tropica* CNB-392	[61]
Polyketide	SS-228 Y	*Chainia* sp. SS-228	[62, 63]
Polyketide	Tetracenomycin D	*Streptomyces* sp. B8005	[32]
Polyketide	Trioxacarcins	*Streptomyces* sp. isolate B8652	[64]
Non-ribosomal peptide	Arenamides	*Salinispora arenicola* CNT-088	[65]
Non-ribosomal peptide	Lucentamycins	*Nocardiopsis lucentensis* CNR-712	[66]
Polyketide/non-ribosomal peptide	Lajollamycin	*Streptomyces nodosus* NPS007994	[67]
Non-ribosomal peptide	Mechercharmycins	*Thermoactinomyces* sp. YM3-251	[68]
Non-ribosomal peptide	Piperazimycins	*Streptomyces* sp. CNQ-593	[69]
Non-ribosomal peptide	Proximicins	*Verrucosispora* sp. MG-37 *Verrucosispora maris* AB-18-032	[70, 71, 72]
Polyketide/non-ribosomal peptide	Salinosporamides	*Salinispora tropica* CNB-392 *Salinispora tropica* CNB-440 *Salinispora tropica* CNB-476	[61, 73, 74, 75, 76]
Non-ribosomal peptide	Thiocoraline	*Micromonospora* sp. L-13-ACM2-092	[77, 78]
Isoprenoid	4a,8a-dimethyl-6-(2-methylpropenyloxy)-3,4,4a,4b,5,6,8a,9-octahydro-1H-phenanthren-2-one	*Actinobacterium* sp. MS1/7	[79]
Isoprenoid	Altemicidin	*Streptomyces sioyaensis* SA-1758	[80, 81]
Isoprenoid	Chlorinated dihydroquinones	Actinomycete isolate CNQ-525	[82]
Isoprenoid	Marinones	Actinomycete isolate CNH-099	[83, 84, 85]
Isoprenoid	T-Muurolol	*Streptomyces* sp. M491	[37, 86]
Indolocarbazole	Arcyriaflavin A	Actinomycete sp. Z2039-2	[87]
Indolocarbazole	K252c	Actinomycete strain Z2039-2	[87]
Indolocarbazole	Staurosporins	*Streptomyces* sp. KS3 *Micromonospora* sp. L-31-CLCO-002 *Streptomyces* sp. QD518	[39, 50, 88]
Indolocarbazole	ZHD-0501	*Actinomadura* sp. 007	[89]
Phenazine	1,6-phenazinediol	*Actinomadura* sp. M048	[90]
Phenazine	Iodinin	*Actinomadura* sp. M048	[91]
Pyrroloiminoquinone	Ammosamides	*Streptomyces* sp. CNR-698	[92]
Pyrrolizidine	Bohemamines	*Streptomyces* sp. CNQ-583	[93]
Butenolide	Butenolides	*Streptoverticillium luteoverticillatum* 11014	[94]
Benzoxazole	Caboxamycin	*Streptomyces* sp. NTK 937	[95]
Acetal-lactone	Echinosporins	*Streptomyces albogriseolus* A2002	[96]
Polypyrrole	Marineosins	*Streptomyces* sp. CNQ-617	[97]
Phenoxazin-3-one	Questiomycins	*Actinomadura* sp. M048	[91]
Methylpyridine	Streptokordin	*Streptomyces* sp. KORDI-3238	[54]
Tetrahydropyrrole	Streptopyrrolidine	*Streptomyces* sp. KORDI-3973	[98]
Prodigiosin	Undecylprodigiosin	*Saccharopolyspora* sp. nov.	[99]

**Table 2:** Important derivatives from marine actinomycetes which possess antiangiogenic potential.

Compound	Marine organism Source	Action	Reference
Streptopyrrolidine	Streptomyces sp. KORDI-3973	Inhibition of tube formation in HUVECs	[98]
Cyclo-(L-Pro-L-Met)	*Nocardiopsis* sp. 03N67	Antiangiogenesis activity against human umbilical vein endothelial cells (HUVECs)	[101]
Streptochlorin	Streptomyces strain 04DH110	• Inhibition of in vitro growth of human leukemia K-562 cells with an IC50 of 1.05 μg/mL significantly • Potent antiangiogenic agent by inducing ROS-mediated apoptosis and inhibits TNF-α-induced NF-κB activation. • Antiangiogenic potential by downregulating the expression of VEGF.	[27, 28, 29, 30]
Lynamicins	Marinispora sp. NPS12745	• Potent antitumour and antiangiogenic properties • Reduction of resistance mediated by transporter ABCG2	[102, 103]
Marizomib	*Salinispora tropica*	Potential anticancer agent and is currently undergoing Phase-I clinical trial.	[8, 104]
Thiocoraline	Micromonospora sp. L-13-ACM2-092	• Potent antitumour activity against melanoma MEL288, human lung adenocarcinoma A549, and murine leukemia P388	[77]

## 3 Zebrafish- a suitable model for angiogenesis research

Zebrafish model is widely used in angiogenesis study as the circulation starts after 24 hours post-fertilization (hpf), and the vascular system bears a strong similarity to that of humans. In the early embryonic development, blood vessels and organ formation can be easily visualized in the transparent embryos and larvae of both wildtype and transgenic species making it a viable model for angiogenesis research [105]. Therefore, this advantage of zebrafish plays an important role in studying tumour angiogenesis, which is crucial for cancer progression and metastasis and also serves as targets for antitumour therapeutics. Staining of vascular endothelial cells of zebrafish by a fluorescent protein can render the observation of newly formed blood vessels in the earliest tumour progressive stage. Zebrafish also serves as a tumour metastasis model; due to its transparent embryos and larvae the metastasizing tumour cells can be exactly traced by the fluorescent-stained tumour cells at the cellular level [106]. Furthermore, the large clutch size of embryos and inexpensiveness of zebrafish make them easily amenable for the large-scale drug screen in antiangiogenic drug discovery and efficacy.

### 3.1 Zebrafish transgenic models in tumour angiogenesis

**Figure 5:**
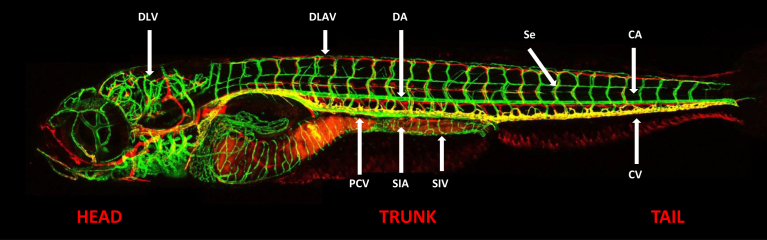
Confocal fluorescence imaging shows blood vessels in green and lymphatics in red. (Adapted from Okuda et al., 2012 [107]). [Abbreviations: DLV- dorsal longitudinal vein, DLAV- dorsal longitudinal anastomotic vessel, DA- dorsal aorta, Se- intersegmental vessel, CA- caudal artery, CV- caudal vein, SIV-subintestinal vein, SIA- supraintestinal artery, PCV-posterior cardinal vein.].

Transgenic technology has improved the characteristic *in vivo* imaging capabilities of zebrafish larvae and embryos. A dissecting microscope is sufficient to visualize the blood flow and vessel development in early embryos and larvae, yet tissue specific expression of fluorescent proteins is required to study the vasculature in detail (Figure 5) [107]. Phenotypic changes and cell shape abnormalities with live specimens can be studied in detail by confocal microscopy and timelapse imaging techniques and thus, formation of vasculature has been explained with the use of molecular markers in detail, both from the cellular and anatomical point of view [108, 109, 127]. Based on gene-specific promoters, transgenic zebrafish mutant lines were developed with vascular-specific phenotypes and both heterologous and autologous promoters have been shown to work. Zebrafish transgenic mutant lines which have been developed to study the vasculature is given in Table 3. The promoter closely similar to mammalian species was used previously; before the availability of whole genome sequence of zebrafish [128].

**Table 3:** **Transgenic zebrafish lines developed to study and visualize the vasculature**.

Line	Expression	Gene	Reference
*Tg(dll4:EGFP)*	Endothelial cells	*Notch ligand*	[110]
*Tg(Tie2:eGFP)*	Endothelial cells	*Tie-2 receptor tyrosine kinase*	[111]
*TgBAC(dll4:GAL4FF)*	Endothelial cells	*Notch ligand*	[112]
*Tg(fli:eGFP)y1*	Endothelial cells, cytoplasmic	*Transcription factor Fli-1*	[113]
*Tg(fli1:neGFP)y7*	Endothelial cells, nuclear	*Transcription factor Fli-1*	[114]
*Tg(5xUAS:cdh5-EGFP)*	Pan-endothelial	*VE-cadherin*	[115]
*Tg(flt4:YFP)*	Pan-endothelial	*Vegfr3*	[116]
*TgBAC(cdh5:Citrine)*	Pan- endothelial	*VE-cadherin*	[117]
*TgBAC(cdh5:GAL4FF)*	Pan- endothelial	*VE-cadherin*	[118]
*TgBAC(flt4:Citrine)*	Pan-endothelial	*Vegfr3*	[119]
*Tg(kdr.eGFP)s843*	Angioblast/endothelial precursors	*Vegfr2/flk1/kdr/Vegfr4*	[120]
*Tg(kdr:G-RCFP)*	Angioblast/endothelial precursors	*Vegfr2/flk1/kdr*	[121]
*Tg(gata1:dsRed)sd2*	Blood cells	*Transcription factor GATA-1*	[122]
*Tg(gata2:eGFP)*	Blood cells	*Transcription factor GATA-2*	[122]
*Tg(hsp70l:canotch3-EGFP)*	Perivascular	*Notch3 intracellular domain*	[123]
*Tg(gata1:GFP)*	Erythroid lineage	*Transcription factor GATA-1*	[124]
*Tg(-7.8gata4:GFP)ae3*	Endocardial and myocardial cells	*Transcription factor GATA-4*	[125]
*Tg(my17:eGFP)*	Myocardial cells	*Cardiac myosin light chain 2*	[126]

Adapted from Baldessari and Mione (2008), Kamei et al. (2010) and Schuermann et al. (2014).

Molecular traces have been employed to study the formation of vasculature in zebrafish, during the embryonic development and thus the vascular anatomy has been well documented which has proven to share quiet a high percentage of resemblance with higher order vertebrates [108, 129, 130]. Strategy involving the injection of microspheres, and their fluorescence detection after lumenization and anastomosis of the vascular network is complete [131] and this has been used to analyze the formation of lymphatic and blood vasculature in zebrafish [132]. Individual cell growth during the formation of vasculature has been attained by using transgenic zebrafish lines and therefore, fluorescent endothelial cell markers are employed to increase the possibility to visualize the migratory and proliferative behaviors of single cells, and various other cell types during the embryo-to-larva transition. Two different cell types were observed simultaneously by combining transgenic lines expressing different fluorescent proteins [113, 133, 134, 135]. Additionally, by using the combination of cell and nuclear membrane specific fluorescent tags, researchers have reported to study the single cell morphological dynamics in living larvae during vascular development [136]. Zebrafish transgenic lines development has been of a much greater utility in studying induced gene expression and also tissue specific gene expression [137]. Thus, these strategies facilitated the study of the sequence of events taking place during the formation of early circulatory loop in zebrafish embryos. The intersegmental vessels are the important angiogenic vessels, whose development is of a greater importance because of its characteristics and high accessibility feature in the zebrafish embryos and larvae; these vessels emanate from dorsal aorta into the embryonic tail and trunk region, and finally grow into the anastomosing dorsal longitudinal vessels [138]. Experimental analysis of blood vessel development in zebrafish embryogenesis was carried out using two common methods namely immunohistochemistry and *in* *situ* hybridization for the visualization of protein and gene expression. But these methods were not specifically developed to study zebrafish vasculature, but various other protocols and tools are currently available that enable these strategies [139, 140]. Regardless of its popularity and success, the researchers using zebrafish model must also contemplate their work by extending their research on other higher vertebrates or mammalian systems, for further clinical applications in future.

### 3.2 Zebrafish in drug screens

The rationale of zebrafish usage for high-throughput drug screening of marine bioactive compounds as become popular in the past decade as these animals involve only sub-milligram quantities for hit selection and validation and are easily pliable to multi-well plates for the reason that they have small sized embryos and larvae [11, 141, 142]. The quantity of marine bioactive compounds for primary screening purposes is limited and it is yet another disadvantage of rodents in marine drug discovery as they require higher quantity for drug screening. As discussed earlier, optical transparency of zebrafish embryos until 5 days post-fertilization (dpf) aids easy visualization of tissues and organs and this feature, allows researchers to employ zebrafish transgenic lines coupled with fluorescently labeled organs and cells, and to study the vascular patterning by developing assay methods for chemical and genetic screening approaches [143, 144]. Significantly, several small bioactive compounds identified in zebrafish possess anticancer properties and are currently in clinical trial phase [141]. Zebrafish can also be used for phenotype-based drug discovery which allows the identification of small molecules independently of their mode of action [141, 142]. Zebrafish embryos and larvae have been used in drug screening strategies so far and antiangiogenic properties of marine compounds studied in zebrafish model are discussed in Table 4.

**Table 4:** List of marine compounds with antiangiogenic properties studied using zebrafish model.

Compound	Action	Targeted molecules	Reference
Solomonamide A	Antiangiogenesis	ERK1/2 and Akt phosphorylation	[145]
Catunaregin	Antiangiogenesis	Modulating phosphorylation of Akt and eNOS	[146]
Somocystinamide A	Antiangiogenesis by inhibiting tube formation of endothelial cells	Caspase-8-expressing tumours	[147]
Stellettin B	Decreased blood vessel formation in developmental zebrafish	VEGF transcriptional expression	[148]
Crambescidin 816	Antitumour effect	Caspase-3 cleavage and activation.	[149]
Bromophenol BDDE	Antiangiogenesisby inhibiting sub-intestinal vessel formation	VEGF/VEGFR	[150]

### 3.3 Zebrafish Xenograft model

Xenografting is a pre-clinical tool used by researches in the recent times to evaluate drug responses and to study tumour metastasis [151]. Zebrafish is established as an efficient model for human tumour xenotransplantation (XT), specifically human leukemias and lymphomas. Absence of adaptive immune system in zebrafish larvae until 28hpf makes them a suitable XT model, with no constraint for immunosuppression. Likewise, the zebrafish XT system allows real time observation and imaging of tumour-cell crescendos in a live animal microenvironment. High conservation is observed in the developmental process of hematopoiesis of zebrafish, making it a robust model to study normal and abnormal blood vessel development and disorders especially in blood cancer research. Therefore, zebrafish can be utilized as a pre-clinical screening model to establish patient-derived cancer cell xenotransplantation and develop novel possibilities for personalized medicine. Table 5 gives important xenograft transplantation cancer models in zebrafish. The first studied xenotransplantation of human cells into zebrafish [153] led many researchers to use the zebrafish embryos to establish the factors underlying in the other sides of cancer biology which includes cancer-induced angiogenesis, cancer cell invasion and metastasis [176, 177]; cancer cells interaction with host cell [178]; and screening of drugs [179, 180]. In a recent study using zebrafish xenografted model (CDX), antiangiogenic effectiveness of ramucirumab, apatinib, regorafenib and cabozantinib was evaluated for the intersegmental vessels (ISVs) and subintestinal veins (SIVs) formation, in which all the four drugs exhibited antiangiogenic potential in the Tg (fli-1: EGFP) zebrafish embryos [181]. Significantly, the laboratory observation of developing a zebrafish tumour model and its response to chemotherapeutics is comparable to mouse xenograft models [182]. With these features, zebrafish can also be considered as a vital XT model to study and identify marine bioactive molecules.

**Table 5:** **List of Human Cancer Xenograft transplantation models in zebrafish**.

Tumours	Transplant stage	Site of injection	Observation	Reference
Melanoma and colorectal cancer (both murine)	48 hpf	Yolk sac, hind brain ventricle	Inhibition of vascularization by VEGFR2 inhibitor - SU5416.	[152]
Melanoma, Uveal melanoma	Blastula 48hpf	Blastodisc Yolk sac	• Studied tumor cell plasticity and investigated tumor microenvironment interactions. • Large scale drug screening and drug discovery	[153] [154]
Prostate Cancer (androgen dependent and independent)	48 hpf	Yolk sac	• Silencing of tyrosine kinase SYK prevented cancer cell dissemination. • Xenograft using LNCaP in zebrafish treated with exogenous testosterone - increased cancer cell proliferation	[155] [156]
Colorectal cancer	48 hpf	Yolk sac	• Activation of by intrinsic apoptotic signaling by Marine guanidine alkaloids in tumour regression. • Efficacy of Bromelain in tumour regression.	[149] [157]
Pancreatic cancer	48 hpf	Yolk sac	Evaluation of tumour cell invasion and micrometastasis with transgenic zebrafish	[158]
Breast cancer	48 hpf	Yolk sac Duct of Cuvier	• Patient-derived material (PDX)model in bone metastasis research • Role of SOX2 interaction with AKT signalling in breast cancer.	[159] [160]
Breast cancer, non-invasive and metastatic	48 hpf	Duct of Cuvier	TGF-β receptor kinase inhibitors for blocking and inhibiting TGF-β signaling.	[161]
Retinoblastoma	48hpf	Vitreous cavity	Orthotopic zebrafish model to understand the invasive and metastatic nature of retinoblastoma	[162]
Glioblastoma	52 hpf	Yolk sack; brain	• Changes in the cell heterogeneity after treatment with chemotherapy on tumour. • Model for detection of BBB (Blood-Brain Barrier) penetration of TNB. • RECQ1 helicase, a promising molecular target in the glioblastomatherapy and high throughput screening	[163] [164] [165]
Gastrointestinal tumours pancreas, stomach, colon	48 hpf	Yolk sac; liver	Inhibition of growth and metastasis in xenografted cells by targeting EGFR and its downstream signing molecules AKT/ERK by Triphala	[166]
Oral squamous cell carcinoma	48 hpf	Yolk sac	Induction of apoptosis by Sandensolide in Oral cancer.	[167]
Non-small-cell lung cancer (NSCLC)	48 hpf	Yolk sac	• Bevacizumab, endostar and apatinib effects and its toxicity were analyzed.	[168]
Ewing sarcoma (EWS)	35 dpf 48 hpf	Yolk sac Eye vessels	Nutlin-3, a tp53 activator, and YK-4-279, a EWSR1–ETS inhibitor as a Combinational therapy was studied.	[169]
MM, Waldenstrom's macroglobulinemia	48hpf	Yolk sac Pericardium	Drug efficacy and sensitivity was analysed using zebrafish PDX. Progression of cancer by cell dissemination and homing to bone marrow were investigated.	[170]
AML	48 hpf	PC vein	Inhibitory effect of imatinib and other antileukemic drugs.	[171]
Glioblastoma, melanoma, breast cancer, RMS	Adult	Peri-ocular muscle	A double mutant immunodeficient zebrafish to study cancer xenotransplantation.	[172]
MM cells from plasma MM cells from bone marrow	48 hpf	Yolk sac Pericardium	Drug sensitivity or resistance were investigated using zebrafish model.	[173]
AML, HCC	48 hpf Adult	Yolk sac, Trunk near dorsal aorta; heart	Treatment with busulfan successfully enabled xenograft AML cells and HCC cells into adult zebrafish	[174]
CML, HCC, prostate cancer	48 hpf Adult	Yolk sac Trunk near dorsal aorta	Model for xenotransplantation and drug screening by introducing cancer stem-like cells.	[175]

## 4 Summary and conclusion

The established angiogenic inhibitors or small bioactive compounds from marine symbiotic actinomycetes provide hope for reducing the morbidity and mortality from metastatic cancers and other carcinomas. Though, it is reported to have successful results with the use of established antiagiogenic drugs which have entered clinical trials, long term survival benefits in cancer patients can be achieved by combination therapy by combining small molecules with chemotherapy or radiation therapy. The neovascularization of cancer tissue as well as the growth of the tumour can be repressed by the use of angiogenesis-suppressors and thus might be helpful in the treatment of cancer and, in particular few bioactive compounds produced by genus *Streptomyces*, serves as a source of numerous antitumour drugs. As marine system consists of enormous beneficial microbes, it is important to take into account for drug discovery as there are innumerable compounds with novel structural diversity which are yet to be discovered from marine actinomycetes. Antiangiogenic marine bioactive compounds have been extensively found successful in cell lines study and rodent models, whereas their usage in zebrafish is still in emergence stage. Therefore, a most potential and successful animal model is required to study the novel drug efficacy in a cost-effective manner. As we discussed in detail above using zebrafish in marine drug discovery, they are already proven model in angiogenesis research, which helps us to identify and discover novel anticancer/antiangiogenic compounds from marine actinobacteria.

## Declarations

### Author contribution statement

All authors listed have significantly contributed to the development and the writing of this article.

### Funding statement

This work was supported by 10.13039/501100006143Department of Science and Technology (DST) - INSPIRE [DST/INSPIRE/04/2018/003392] and DST- Science & Engineering Research Board (SERB) - National Postdoctoral Fellowship [File Number: PDF/2016/003879].

### Data availability statement

No data was used for the research described in the article.

### Declaration of interests statement

The authors declare no conflict of interest.

### Additional information

No additional information is available for this paper.
